# Effect of Serial Home-Based Exercise Immediately after Latissimus Dorsi Reconstruction in Patients with Breast Cancer

**DOI:** 10.3390/healthcare10091760

**Published:** 2022-09-13

**Authors:** Eunhee Park, Joon Seok Lee, Ho Yong Park, Jung Dug Yang, Tae-Du Jung

**Affiliations:** 1 Department of Rehabilitation Medicine, School of Medicine, Kyungpook National University, Daegu 41944, Korea; 2 Department of Rehabilitation Medicine, Kyungpook National University Chilgok Hospital, Daegu 41404, Korea; 3 Department of Plastic and Reconstructive Surgery, School of Medicine, Kyungpook National University, Daegu 41944, Korea; 4 Department of Plastic and Reconstructive Surgery, Kyungpook National University Chilgok Hospital, Daegu 41404, Korea; 5 Department of Surgery, School of Medicine, Kyungpook National University, Daegu 41944, Korea; 6 Department of Surgery, Kyungpook National University Chilgok Hospital, Daegu 41404, Korea

**Keywords:** breast neoplasm, mammaplasty, recovery of function, exercise

## Abstract

Purpose: This study investigated the effects of a serial home-based exercise program in the affected upper extremity immediately after latissimus dorsi (LD) flap reconstruction with mastectomy in order to improve the functional impairment and quality of life in breast cancer survivors. Methods: Patients with breast cancer scheduled for a mastectomy immediately followed by autologous LD flap reconstruction surgery were enrolled. Forty-five patients were included as an intervention group who received a serial home-based exercise program with stretching and strengthening for upper extremities preoperatively (T0), and 2 weeks (T1), 6 weeks (T2), and 3 months (T3) postoperatively. Thirty-five patients were included as the control group. We evaluated the range of movement in the shoulder at T0, T1, T2, T3, 6 months (T4), and 12 months (T5) postoperatively. We also evaluated the disability of the upper extremity using disabilities of the arm, shoulder, and hand (DASH) questionnaire and quality of life using the 36-Item Short-Form Health Survey (SF-36) at T0, T3, T4, and T5. Results: There were significant differences in interaction effects between time and shoulder flexion and internal and external rotation. Post hoc, the intervention group showed more improvement of movement in internal rotation at T2 and T5 and external rotation at T2, T3, and T4. Furthermore, there were significant differences in interaction effects between DASH scores and time in the two groups. Post hoc, there were significantly lower DASH scores at T3, T4, and T5 in the intervention group. There were significant differences in interaction effects of physical role functioning, vitality, and mental health scores of SF-36 and time in the two groups. Post hoc, physical role functioning scores at T3 and T4 and vitality and mental health scores at T3 were elevated in the intervention group. Conclusion: A serial home-based exercise after LD flap reconstruction is effective for the rehabilitation of the affected upper extremity and enhances the quality of life.

## 1. Introduction

Breast reconstruction is widely considered after surgical treatment of breast cancer [1]. Among the many reconstruction methods, immediate autologous tissue transfer of the latissimus dorsi (LD) flap surgery is commonly used in patients with breast cancer who have small or moderate-sized breasts [2].

Previous studies reported functional recovery of the affected upper extremity immediately after LD flap reconstruction. A review study demonstrated that LD flap reconstruction affects shoulder dysfunction, which is minimally impaired at 3 months after surgery [3]. In addition, other review studies reported reductions in range of motion and strength in the shoulder joint, and these reductions were resolved within 6 to 12 months postoperatively [4,5]. Furthermore, a previous study reported that patients who received LD flap reconstruction recovered muscle strength and range of motion in the shoulder joint at 12 months postoperatively [6]. However, these studies describe spontaneous functional recovery after LD flap reconstruction but not the effects of additional exercise on functional recovery of the upper extremity after surgery.

The effects of exercise on the recovery of shoulder function after LD flap surgery is uncertain. Oliveira et al. [7] reported that patients with mastectomy and LD flap underwent three sessions of physical therapy in a hospital for four weeks postoperatively compared with mastectomy alone. They showed that up to a year following surgery, immediate LD flap and postoperative physical therapy had no impact on the shoulder functions or postsurgical complications. In Button et al. [8], all patients were given only one home-based exercise after LD flap surgery, and there were no subjects that did not conduct the exercise. In addition, they did not demonstrate details about the type, duration, and frequency of the home-based exercise program. Furthermore, in the study by Glassey et al. [9], a shoulder exercise program 2 days after surgery resulted in more effective shoulder movement and improvement of disability compared with the preoperative state. However, these studies did not compare the exercise intervention group with a non-exercise control group. In addition, these studies did not confirm the effect of serial exercise through the postoperative periods.

Therefore, the purpose of this study is to investigate the effect of serial home-based exercise immediately after LD flap surgery in patients with breast cancer compared to those patients who did not exercise. We hypothesized that patients with serial home-based exercise would have better shoulder movement, less disability, and a higher quality of life than patients without exercise.

## 2. Methods

### 2.1. Participants

This study is a non-randomized and historically controlled study to determine the effects of serial home-based exercise compared with non-exercise after the same operation. This study included patients with diagnosed unilateral breast cancer who underwent immediate LD flap reconstruction after mastectomy surgery, in an age range of 30–60 years at the time of surgery, and who also attended four serial home-based exercise programs. The study excluded patients with diagnosed advanced stage IV breast cancer with a history of neurologic disorders or musculoskeletal problems of the trunk and the upper extremity (e.g., adhesive capsulitis, lateral epicondylitis) or who were unable to answer the self-questionnaire due to cognitive impairment. One hundred twenty-six patients who received the first exercise session for home-based exercise programs from May 2018 to December 2020 were eligible. Two patients with bilateral breast cancer did not meet the inclusion criteria. Forty patients were eliminated for exclusion criteria. Eighty-four patients attend all exercise education sessions. Thirty-nine patients failed to attend follow-up until 12 months after surgery. Finally, 45 patients were included and analyzed as the intervention group. In addition, 35 patients were included in previously published data as the control group from 2011 to 2013 [6]. The control group was enrolled with the same inclusion and exclusion criteria, except for attending home-based exercise programs in this study (Supplementary Figure S1).

Written informed consent was obtained from all participants in the intervention group, and ethical approval was provided by the Institutional Review Board (IRB) of Kyungpook National University Chilgok Hospital (IRB No. 2018-04-002).

### 2.2. Breast Reconstruction Using Latissimus Dorsi Flap

A patient who was diagnosed with unilateral breast cancer underwent a mastectomy in the supine position by a breast surgeon and then was changed to a decubitus position for immediate extended LD flap reconstruction. After this procedure was completed, a plastic surgeon performed humoral detachment of the LD muscle and transferred the flap using axillary tunneling to the defective breast area. When breast volume was insufficient, a small implant was added to reconstruct the breast with a shape similar to a healthy breast.

### 2.3. Serial Home-Based Exercise Program

Patients who were included in the intervention group received a serial home-based exercise program education by a physiotherapist or an expert physician at the Department of Rehabilitation Medicine as a preoperative outpatient (T0), followed at 2 weeks (T1), 6 weeks (T2), and 3 months (T3) postoperatively. The serial home-based exercise program, which was designed by a physician (E.P), consisted of stretching exercises of the back, chest, and shoulder joints and strengthening of the shoulder girdle muscles and upper extremity muscles. Each patient was taught the first exercise program, which was performed immediately after surgery, during a preoperative outpatient visit to the Department of Rehabilitation Medicine (T0). It consisted of a shoulder roll, shrug, and limitation of shoulder movement on the forward flexion for 90° and on abduction for 45° when the breast drain is in place (Supplementary Figure S2a). Each patient was taught the second exercise program at T1, which included shoulder circling, forward lifting on the wall as high as possible, lifting to the side on the wall as high as possible, scapular retraction as tolerated, shoulder external and internal rotation during adduction and elbow 90° flexion, and isometric shoulder abduction (Supplementary Figure S2b). Each patient was taught the third exercise program at T2, which included shoulder abduction, lateral trunk stretching during shoulder 180° abduction for relaxation of the LD muscle, anterior trunk stretching for relaxation of the pectoralis muscle, shoulder forward lifting using a towel, isotonic concentric strengthening of shoulder external and internal rotation, and abduction using a light dumbbell (Supplementary Figure S2c). Finally, each patient was taught the fourth exercise program at T3, which included scapular protraction stretching for relaxation of the LD muscle, anterior trunk stretching using the wall for relaxation of the pectoralis muscle, isotonic eccentric shoulder external and internal rotation, and extension with scapular depression using an elastic resistance band (Supplementary Figure S2d). A physiotherapist or a physician instructed the patient how to exercise, and then the patient performed the same exercise under supervision at T0, T1, T2, and T3.

### 2.4. Functional Assessments

A physiotherapist or a physician evaluated functional assessments when a patient visited the outpatient clinic of the Department of Rehabilitation Medicine.

#### 2.4.1. Active Range of Motion in the Shoulder Joint

The active range of motion (ROM) of the shoulder is a useful assessment of the integrity of a shoulder joint after surgery. ROM is measured with a goniometer in the standard anatomic position. ROM of shoulder flexion and abduction was measured in the upright position. The normal range for shoulder flexion and abduction is 0–180°. In the supine position at 90° of shoulder abduction and 90° of elbow flexion, the ROM of shoulder external and internal rotation was measured. The normal range for shoulder external and internal rotation is 0–90° [10]. We evaluated the range of movement in shoulder at T0, T1, T2, T3, 6 months (T4), and 12 months (T5) postoperatively.

#### 2.4.2. Disability of the Upper Extremity

Assessment of upper extremity function was performed using the disabilities of the arm, shoulder, and hand (DASH) questionnaire. It consists of 30 items, each with five responses; 21 items assess the degree of difficulty in performing different physical activities, 6 items assess symptoms, and 3 items assess psychosocial effects. A score of 0 indicates no disability, and a score of 100 indicates complete disability. We used the Korean version of the questionnaire for its reliability and validity in measuring the upper extremity dysfunction, which has been proven [11]. We evaluated the DASH questionnaire at T0, T3, T4, and T5.

#### 2.4.3. Quality of Life Using the 36-Item Short-Form Health Survey

The 36-Item Short-Form Health Survey (SF-36) is a widely used and patient-reported measure of health status [12]. It comprises four physical domain subscales: physical functioning (PF), role functioning-physical (RP), bodily pain (BP), and general health (GH). These four components are combined in a physical component summary scale (PCS). The survey also has four mental domain subscales: vitality (VT), social functioning (SF), role functioning-emotional (RE), and mental health (MH), which are combined in a mental component summary scale (MCS). We used the Korean version of the SF-36 for its reliability and validity, which has been proven [13]. We evaluated SF-36 at T0, T3, T4, and T5.

### 2.5. Statistical Analysis

All statistical analyses were performed using SPSS 23.0 (SPSS Inc., Chicago, IL, USA). It was determined that assessments were normally distributed according to the Shapiro–Wilk test. We performed a *t*-test to compare the baseline characteristics of patients according to two groups at T0 baseline. Furthermore, repeated measures analysis of variance (RMANOVA) with the Bonferroni post hoc test was performed to evaluate the interaction effects of time (T0, T1, T2, T3, T4, and T5) and the ROM of the shoulder in the group (control and intervention). In addition, the RMANOVA with Bonferroni post hoc test was performed to evaluate the interaction effects of time (T0, T3, T4, and T5) and scores of DASH and SF-36 in the group (control and intervention).

## 3. Results

Table 1 describes the general and clinical characteristics of 80 patients. There were no significant differences in the distribution of sex, age, the clinical status of breast cancer, ROM of the shoulder, DASH score, and SF-36 score between the two groups at T0.

When comparing the interaction effect of time and group in ROM of the shoulder, there was a significant time and group interaction effect of shoulder flexion (*F* = 3.127, *p* = 0.017). In Bonferroni post hoc analysis, there was a significantly higher value of shoulder flexion at T2 (6 weeks after surgery) in the intervention group compared with those in the control group (95% confidence interval [CI]: 0.86–17.43, *p* = 0.031). Furthermore, there was a significant time and group interaction effect of shoulder internal rotation (*F* = 2.604, *p* = 0.038). In post hoc analysis, there were significantly higher values of shoulder internal rotation at T2 (6 weeks after surgery, CI: 4.13–17.57, *p* = 0.002) and T5 (12 months after surgery, CI: 0.90–9.98, *p* = 0.020) in the intervention group compared with the control group. In addition, there was a significant time and group interaction effect of shoulder external rotation (*F* = 2.770, *p* = 0.047). In post hoc analysis, there were significantly higher values of shoulder external rotation at T2 (6 weeks after surgery, CI: 7.02~18.12, *p* = 0.000), T3 (3 months after surgery, CI: 6.09–16.93, *p* = 0.000), and T4 (6 months after surgery, CI: 3.49–15.88, *p* = 0.003) in the intervention group compared with the control group (Table 2).

**Figure 1:**
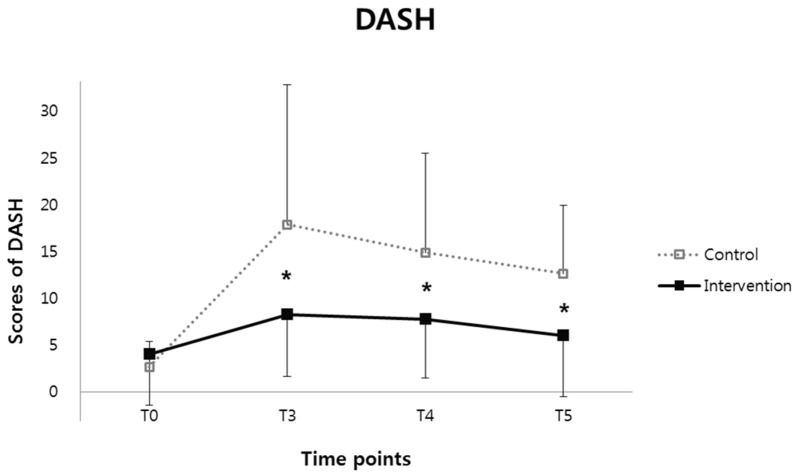
The disability of the upper extremity using disabilities of the arm, shoulder, and hand (DASH) questionnaire and quality of life using a 36-Item Short-Form Health Survey (SF-36) at the preoperative stage (T0), and then 3 months (T3), 6 months (T4), and 12 months (T5) postoperatively. There were significantly lower DASH scores in the intervention group compared with those in the control group with Bonferroni post hoc analysis (* *p* < 0.05).

There was a significant time and group interaction effect of the DASH score (*F* = 5.809, *p* = 0.005). There were significantly lower DASH scores at T3 (3 months after surgery, CI: −17.52–−1.67, *p* = 0.019), T4 (6 months after surgery, CI: −13.21–−0.96, *p* =0.025), and T5 (12 months after surgery, CI: −11.63~−1.55, *p* = 0.012) in the intervention group compared with those in the control group with Bonferroni post hoc analysis (Figure 1 and Table 3).

Table 4 shows the values in the subscale of SF-36 at each time point in both groups. There was a significant time and group interaction effect of RP score (*F* = 3.064, *p* = 0.032). In Bonferroni post hoc analysis, there were significantly higher values of RP at T3 (3 months after surgery, CI: 6.60–60.66, *p* = 0.016) and T4 (6 months after surgery, CI: 0.88–48.45, *p* = 0.043) in the intervention group compared with the control group. In addition, there was a significant time and group interaction effect of VT score (*F* = 3.128, *p* = 0.029). In Bonferroni post hoc analysis, there was a significantly higher value of VT at T3 (3 months after surgery, CI: 0.09–25.36, *p* = 0.048) in the intervention group compared with the control group. Furthermore, there was a significant time and group interaction effect of MH score (*F* = 2.927, *p* = 0.049). There was a significantly higher value of MH at T3 (3 months after surgery, CI: 1.20–24.21, *p* = 0.031) in the intervention group compared with the control group.

## 4. Discussion

Breast cancer survivors who received the serial home-based exercise at 3 months after LD flap reconstruction showed more improvement of shoulder ROM, disability of the upper extremity, and quality of life than the group that did not. In the intervention group, there was a significant improvement in shoulder internal rotation at 6 weeks and 12 months after surgery and external rotation at 6 weeks, 3 months, and 6 months after surgery. In addition, there was an improvement in the upper extremity function at 3 months, 6 months, and 12 months after surgery. Furthermore, the serial home-based program proved to be effective in improving the quality of life at 3 months and 6 months after surgery. This was the first study that demonstrated the effect of serial home-based exercise after LD flap reconstruction compared with those who did not exercise.

The LD muscle originates on an aponeurosis from the lower thoracic and lumbar vertebra, the iliac crest, and the sacrum and inserts on the inferior angle of the scapula and the intertubercular groove of the humerus [14]. The LD muscle affects the internal rotation, adduction, and extension of the shoulder joint [15]. Previous literature supported that other synergistic muscles, including the teres major muscle in the shoulder joint, may compensate for the loss of function of the LD muscle [14,16]. Therefore, functional impairments spontaneously recovered until a year after LD flap reconstruction [4,5,6]. We focused on serial home-based exercise being effective in early recovery of shoulder joint muscles, including the remaining LD muscle, compared to spontaneous recovery after LD flap reconstruction. Our serial home-based exercise program affected the faster functional recovery of shoulder function compared to spontaneous recovery after LD flap construction.

Our results demonstrated that patients who participated in a serial home-based exercise program after LD flap were more satisfied with the physical domain of quality of life at 3 and 6 months after surgery than those who did not exercise. Patients were taught serial home-based exercises of the upper extremity preoperatively to 3 months postoperatively. The RP subscale, which is a role limitation because of the PF of the SF-36, includes difficulties in work or daily life activities caused by physical health problems over the past month. This resulted in more satisfaction in the intervention group for 6 months postoperatively than in the control group. However, there was no significant difference in the two groups at 12 months postoperative. Further study with additional home-based exercise at 12 months after surgery would be needed to confirm whether patients can maintain the effect of a current home-based exercise program through several longitudinal years.

In our study, patients who performed serial home-based exercise improved in the mental domain of SF-36 compared to patients who did not perform the exercise at 3 months after surgery. It is thought that the improvement of vitality through performing exercise may have affected the improvement of mental health. However, these effects did not last until a year after surgery. Indeed, nearly 50% of breast cancer survivors have suffered from mood changes such as depression, anxiety, or both a year after diagnosis [17]. The scores of the mental domain of SF-36 from exercise may be negligible compared to the effects from mood changes. In order to exclude mood effects in the SF-36, further study is needed to evaluate psychological assessments, such as Beck depression and anxiety inventories, as well as physical assessments.

There are several limitations. First, our study conducted a non-randomized controlled study with historical data. A randomized controlled clinical trial with a concurrent control arm is the optimal way to minimize bias when evaluating the effects of home-based exercise after surgery. Further study is needed to design a randomized controlled study. Second, this study is limited by a small sample size, which may lead to higher variability and bias. Further study is needed to qualify sample size. Third, we did not directly check patients’ satisfaction or compliance in performing serial home-based exercises in the intervention group. For standardized protocol of rehabilitation after LD flap reconstruction, further study is needed to determine the relationship between the dose of home-based exercise and the effectiveness of shoulder ROM, improvement in disability, and quality of life.

## 5. Conclusions

In conclusion, patients with breast cancer who received and participated in the serial home-based exercise program after LD flap reconstruction had a more effective recovery and improvement in disability of the upper extremity up to a year after surgery and improvement in the quality of life for several months postoperative compared to those not receiving exercise after LD flap reconstruction.

## Figures and Tables

**Table 1:** Clinical characteristics of participants.

	Control Group (*N* = 35)	Intervention Group (*N* = 45)	*p* Value
Age, years, mean ± SD	43.6 ± 5.9	46.0 ± 6.0	0.077
Breast cancer			
Tumor location, N (%)			0.222
Right	18 (51.4)	17 (37.8)	
Left	17 (48.6)	28 (62.2)	
Tumor type, N (%)			0.225
DCIS	6 (17.1)	15 (33.3)	
ILC	1 (2.9)	2 (4.5)	
IDC	28 (80.0)	28 (62.2)	
Cancer stage, N (%)			0.521
Stage 0	3 (8.6)	7 (15.6)	
Stage I	14 (40.0)	19 (42.2)	
Stage II	17 (48.6)	19 (42.2)	
Stage III	1 (2.8)	0 (0.0)	
Lymph node dissection, N (%)			0.050
SLNB	24 (68.6)	39 (86.7)	
ALND	11 (31.4)	6 (13.3)	
Adjuvant chemotherapy, N (%)	27 (77.1)	27 (60.0)	0.104
Adjuvant radiotherapy, N (%)	15 (42.9)	28 (62.2)	0.085
Adjuvant hormone therapy, N (%)			0.604
Tamoxifen	27 (77.1)	36 (80.0)	
Letrozole	5 (14.3)	3 (6.7)	

N, number of patients; LD, latissimus dorsi flap; mean ± SD, mean ± standard deviation; ALND, axillar lymph node dissection; BCS, breast conservative surgery; DCIS, ductal carcinoma in situ; IDC, invasive ductal carcinoma; ILC, invasive lobular carcinoma; SLNB, sentinel lymph node biopsy.

**Table 2:** The values in range of motion of shoulder.

	Control Group						Intervention Group						*p* Value
	T0	T1	T2	T3	T4	T5	T0	T1	T2	T3	T4	T5	
Flexion	179.0 ± 2.4	141.7 ± 22.7	162.2 ± 13.7	171.3 ± 7.2	177.7 ± 3.4	177.4 ± 5.6	178.3 ± 9.0	156.3 ± 22.2	170.9 ± 12.7 ^†^	175.2 ± 10.3	175.0 ± 14.4	179.2 ± 2.2	0.043 *
Abduction	178.8 ± 2.8	142.4 ± 28.4	164.3 ± 18.3	172.9 ± 8.1	177.9 ± 3.3	179.2 ± 2.3	179.3 ± 3.3	150.5 ± 34.9	170.0 ± 16.0	176.4 ± 12.9	175.5 ± 14.8	179.4 ± 2.4	0.160
Internal Rotation	82.6 ± 9.7	75.5 ± 13.1	76.4 ± 12.2	80.9 ± 8.2	82.7 ± 8.7	83.8 ± 9.1	87.6 ± 9.0	76.5 ± 15.8	88.6 ± 6.4 ^†^	86.1 ± 13.0	84.3 ± 13.6	89.8 ± 0.9 ^†^	0.038 *
External Rotation	81.7 ± 8.5	74.3 ± 12.7	76.6 ± 10.8	77.7 ± 10.6	79.0 ± 12.2	80.8 ± 11.7	86.2 ± 5.8	76.0 ± 15.1	89.8 ± 1.5 ^†^	89.7 ± 1.7 ^†^	89.5 ± 2.3 ^†^	88.0 ± 5.9	0.047 *

Each cell represents mean ± standard deviation (°). T0: pre-operation, T1: 2 weeks after operation, T2: 6 weeks after operation, T3: 3 months after operation, T4: 6 months after operation, T5: 12 months after operation; * represents a significant time and group interaction effect (*p* < 0.05); ^†^ represents a significant higher value in range of motion of shoulder in the intervention group compared with the control group with Bonferroni post hoc analysis (*p* < 0.05).

**Table 3:** The values in disabilities of the arm, shoulder, and hand questionnaire (DASH score).

	Control Group				Intervention Group				*F*	*p* Value
	T0	T3	T4	T5	T0	T3	T4	T5		
DASH	2.7 ± 2.7	17.8 ± 14.9	14.8 ± 10.6	12.6 ± 7.3	4.0 ± 5.5	8.2 ± 6.6 ^†^	7.7 ± 6.3	6.0 ± 6.5 ^†^	5.809	0.005 *

Each cell represents mean ± standard deviation. DASH: disabilities of the arm, shoulder and hand questionnaire, T0: pre-operation, T3: 3 months after operation, T4: 6 months after operation, T5: 12 months after operation; * represents a significant time and group interaction effect (*p* < 0.05); ^†^ represents a significant lower value of DASH score in the intervention group compared with the control group with Bonferroni post hoc analysis (*p* < 0.05).

**Table 4:** The values in the 36-Item Short-Form Health Survey (SF-36 score).

	Control Group				Intervention Group				*F*	*p* Value
	T0	T3	T4	T5	T0	T3	T4	T5		
PF	91.5 ± 15.4	84.1 ± 17.4	83.8 ± 13.7	85.5 ± 14.2	91.2 ± 15.1	83.2 ± 16.9	80.3 ± 18.6	86.9 ± 15.5	1.769	0.174
RP	67.4 ± 39.5	48.9 ± 45.3	58.3 ± 37.4	76.3 ± 36.8	81.7 ± 30.8	61.8 ± 38.6 ^†^	62.5 ± 39.7 ^†^	75.0 ± 33.3	3.064	0.032 *
BP	78.2 ± 18.0	77.3 ± 16.1	75.8 ± 16.4	82.3 ± 15.3	83.1 ± 15.3	72.6 ± 13.6	74.3 ± 19.3	81.9 ± 18.3	0.653	0.583
GH	59.6 ± 22.8	68.6 ± 13.2	61.5 ± 19.3	63.7 ± 21.3	62.1 ± 15.5	50.2 ± 22.5	56.6 ± 18.4	64.0 ± 18.2	0.136	0.938
PCS	50.6 ± 8.0	46.9 ± 8.3	46.8 ± 7.6	48.9 ± 6.6	51.9 ± 6.3	46.6 ± 6.1	46.1 ± 8.0	49.8 ± 7.1	0.516	0.672
VT	55.2 ± 18.6	57.7 ± 21.9	55.7 ± 16.0	61.8 ± 18.6	53.1 ± 18.1	62.8 ± 16.2 ^†^	56.1 ± 15.9	60.2 ± 17.6	3.128	0.029 *
SF	81.5 ± 22.9	80.7 ± 18.8	77.4 ± 21.1	84.9 ± 18.9	88.3 ± 18.2	82.0 ± 16.9	79.3 ± 16.8	90.7 ± 16.1	0.204	0.894
RE	69.6 ± 41.3	62.1 ± 45.2	69.8 ± 37.9	78.9 ± 38.8	77.0 ± 34.7	65.7 ± 43.0	64.0 ± 41.3	75.3 ± 35.3	1.209	0.311
MH	60.2 ± 17.8	63.6 ± 19.3	68.4 ± 16.4	74.3 ± 17.6	64.0 ± 20.3	72.6 ± 15.9 ^†^	68.8 ± 15.2	71.1 ± 17.6	2.927	0.049 *
MCS	39.8 ± 11.1	41.4 ± 11.4	42.9 ± 10.3	46.3 ± 10.8	41.9 ± 11.3	44.8 ± 10.1	42.8 ± 8.2	45.4 ± 9.6	1.150	0.333

Each cell represents mean ± standard deviation. PF: physical functioning, RP: physical role functioning, BP: bodily pain, GH: general health, PCS: physical component summary, VT: vitality, SF: social functioning, RE: emotional role functioning, MH: mental health, MCS: mental component summary, T0: pre-operation, T3: 3 months after operation, T4: 6 months after operation, T5: 12 months after operation; * represents a significant time and group interaction effect (*p* < 0.05); ^†^ represents a significant higher value of subscale SF-36 score in the intervention group compared with the control group with Bonferroni post hoc analysis (*p* < 0.05).

## Data Availability

Not Applicable.

## Supplementary Materials

The following supporting information can be downloaded at: https://www.mdpi.com/article/10.3390/healthcare10091760/s1, Figure S1: The flowchart of enrollment as the intervention group; Figure S2: The serial home-based exercise program. The first exercise was instructed at preoperatively (a), the second exercise was instructed at 2 weeks after surgery (b), the third exercise was instructed at 6 weeks after surgery (c), and the fourth exercise was instructed at 3 months after surgery (d).
