# Supplementary material for: Effect of Serial Home-Based Exercise Immediately after Latissimus Dorsi Reconstruction in Patients with Breast Cancer
Source: Healthcare (Basel). 2022 Sep 13;10(9):1760. doi: 10.3390/healthcare10091760 (PMC9498885; doi:10.3390/healthcare10091760)

Figure S1. The flowchart of enrollment as the intervention group.

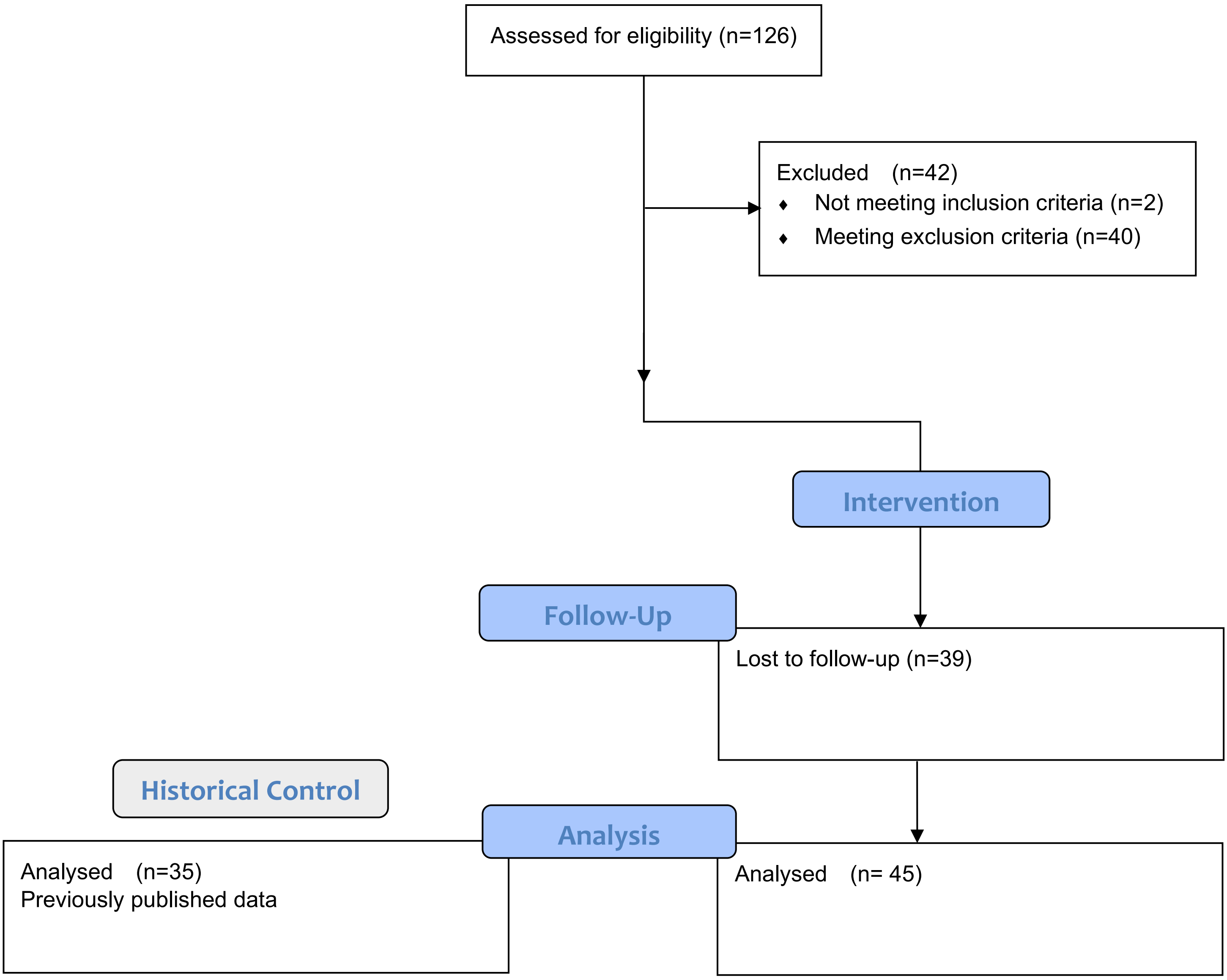

Figure S2. The serial home-based exercise program. The first exercise was instructed at preoperatively (a), the second exercise was instructed at 2 weeks after surgery (b), the third exercise was instructed at 6 weeks after surgery (c), and the fourth exercise was instructed at 3 months after surgery (d).

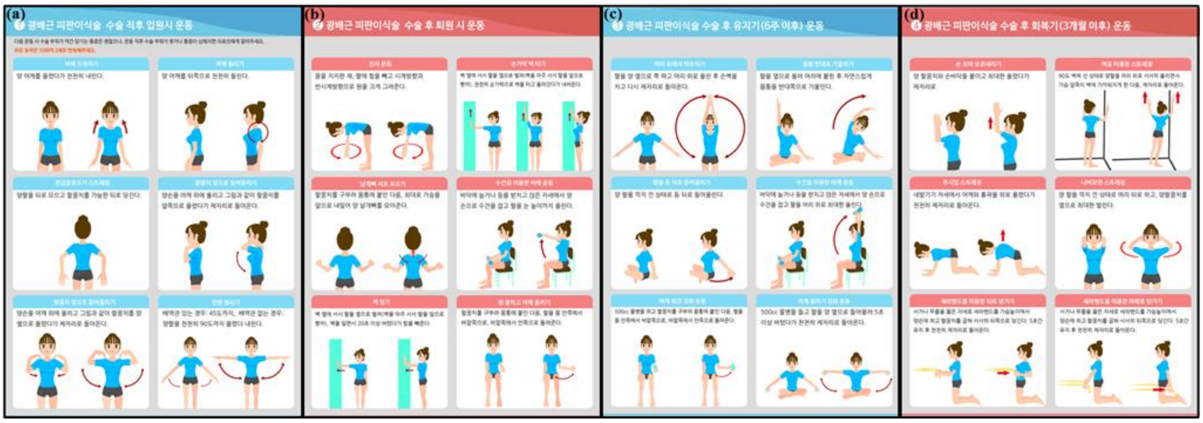

Supplement: Supplementary file 1 [file healthcare-10-01760-s001.zip › healthcare-1870328-supplementary.pdf]
